# Zeolite/Cellulose Acetate (ZCA) in Blend Fiber for Adsorption of Erythromycin Residue From Pharmaceutical Wastewater: Experimental and Theoretical Study

**DOI:** 10.3389/fchem.2021.709600

**Published:** 2021-07-14

**Authors:** Shehdeh Jodeh, Israa Erman, Othman Hamed, Younes Massad, Ghadir Hanbali, Subhi Samhan, Omar Dagdag, Savaş Kaya, Goncagül Serdaroğlu

**Affiliations:** ^1^ Department of Chemistry, Faculty of Science, An-Najah National University, Nablus, Palestine; ^2^ Palestinian Water Authority, Ramallah, Palestine; ^3^ Laboratory of Agroresources, Polymers and Process Engineering (LAPPE), Department of Chemistry, Faculty of Science, Ibn Tofail University, Kenitra, Morocco; ^4^ Department of Pharmacy, Health Services Vocational School, Sivas Cumhuriyet University, Sivas, Turkey; ^5^ Mathematics and Science Education, Sivas Cumhuriyet University, Sivas, Turkey

**Keywords:** pharmaceutical industry, adsorption capacity, wastewater, cellulose, zeolite

## Abstract

The expanding amount of remaining drug substances in wastewater adversely affects both the climate and human well-being. In the current investigation, we developed new cellulose acetic acid derivation/zeolite fiber as an effective technique to eliminate erythromycin (ERY) from wastewater. The number of interchangeable sites in the adsorbent structures and the ratio of ERY to the three adsorbents were identified as the main reasons for the reduction in adsorption as the initial ERY concentrations increased. Additionally, for all adsorbents, the pseudo–second-order modeling showed better fitting for the adsorption than the pseudo–first-order modeling. However, the findings obtained in the pseudo–first-order model were still enough for explaining the sorption kinetics of ERY, showing that the surface displayed all chemisorption and physi-sorption adsorption processes by both adsorbents. The *R* ^2^ for the second order was very close to 1 for the three adsorbents in the case of pseudo–second-order. The adsorption capacity reached 17.76 mg/g. The three adsorbents showed negative values of ΔH, and these values were −6,200, −8,500, and −9600 kJ/mol for zeolite, CA, and ZCA, respectively, and this shows that the adsorption is exothermic. The desorption analysis shows no substantial loss of adsorption site after three trials, indicating higher stability and resilience of the three adsorbents, indicating a strong repeatability of their possible use in adsorption without contaminating the environment. In addition, the chemical attitude and possible donor–acceptor interactions of ERY were assessed by the quantum chemical parameters (QCPs) and NBO analysis performed, at the HF/6-311G** calculations.

## Introduction

One of the most important reasons for the economic growth of developing countries and the expansion of urban areas is a society’s ability to provide fresh water for sanitation and consumption to its population. However, as the population and urbanization increase, so does the release of radioactive materials into the atmosphere and surface water. There are many sources of surface and groundwater contamination, including agricultural, industrial, oil pollution, sewage, and wastewater (Al-Shaalan et al., 2019; El-Zawily et al., 2019; Khan et al., 2019; Chon et al., 2020).

Several water pollution scenarios including the chiral pollution are a serious issue for our health and environment due to the enantioselective biodegradation of the chiral pollutants. It has adverse impact on our society and science. There is a big loss of our economy due to the use of racemic agrochemicals. The most notorious chiral pollutants are pesticides, polychloro biphenyls, polyaromatic hydrocarbons, brominated flame retardants, drugs, and pharmaceuticals (Basheer, 2018a; Basheer and Ali, 2018).

Nowadays, water contamination due to the drugs and pharmaceutical residues is increasing and alarming. These contaminants are called as new emerging pollutants. The contamination due to the new emerging contaminants is of great concern due to their endocrine, hormonal, and genetic disturbance nature (Basheer, 2018b).

In environmental samples such as surface water, groundwater, seawater, soil, and drinking water, pharmaceuticals were found (Arshad et al., 2020; Kiszkiel-Taudul, 2021), so they are referred to as emerging pollutants. The estimated global consumption of pharmaceuticals such as antibiotics is 100,000 to 200,000 tons per annum (Bungau et al., 2020). Based on the chemical properties of the drug, about 5–90% of the absorbed antibiotic doses are excreted by urine or stool as a metabolite or parent compound (Bhowmick et al., 2020). These drugs end up in drainage systems and eventually reach the ecosystem by sewage leakage, discharge of wastewater treatment plant (WWTP) effluents into marine systems, or disposal of unwanted or unfinished medications (Barchiesi et al., 2020). The use of sludge and animal waste as fertilizer in agriculture can also contribute to the degradation of agricultural soils, which can lead to the incorporation of antibiotics into marine environments by leaching into groundwater (Stevens and Jones, 2003).

In recent years, the Environmental Protection Agency (EPA) has been more involved in informing the public about new pollutants of concern (CECs). CECs are a form of pollutant that is commonly found at trace levels in surface and groundwater (i.e., ppb and ppt). Examples of CECs are pesticides, chemicals, anti-infection agents, over-the-counter meds, mechanical synthetics, oil-based synthetic compounds, and others (Farré, 2020). Some of these processes, in particular, lack actual removal procedures, and the by-products generated, such as organochlorine species, may be more toxic than the original compounds (Mery-Araya et al., 2019).

To deal with this wastewater problem, lots of conventional and advanced technologies have been developed (Ali et al., 2018a; Mery-Araya et al., 2019). The conventional water treatments such as oxidation (Ma et al., 2020), electro precipitation, membrane separation, coagulation–flocculation, evaporation, floatation, and ion exchange (Yu et al., 2021) have been largely used, but these are inadequate techniques for water treatments (Tabassum, 2019).

Many approaches have been used and reported for the removal of a variety of pesticides and drugs. Among the different methods, adsorption is the best approach because of several advantages associated with adsorption including time and cost (Ali et al., 2018a; Ali et al., 2018b; Ali et al., 2019).

Erythromycin (ERY) is a natural antibiotic used to treat a variety of bacterial infections. Antibiotics pass into the human body after consistent treatment and ultimately enter inland areas and effluents; there is even a path of environmental degradation in the poultry and livestock breeding industries. Because of the structure of their aromatic ring, ERY molecules are resistant to the environment and difficult to degrade. Several reports (Ma et al., 2020; Yu et al., 2021) have reported the presence of ERY in water and wastewater to be above the average range. As a result, removing ERY residues from wastewater is important.

Zeolite is a crystalline aluminosilicate with well-defined micropore dimensions and a strong crystal lattice form that is environmentally friendly. Zeolite structures are made up of tetrahedral SiO4 and AlO4 groups, and their alumina silica ratio (SAR) determines zeolite polarity (Martucci et al., 2012). Because of their three-dimensional framework, which creates nanometer-sized channels and cages, these materials have a high porosity and a large surface area. The shape of their internal pore structure can have a direct impact on their adsorption selectivity against host molecules, which is one of their distinguishing features (Zide et al., 2018).

Cellulose acetate is an excellent candidate for use as a polymer matrix because it can be easily molded into a variety of shapes and because its hydrophilic surfaces can improve the mobility of aqueous solutions to the surface of hybrid materials (Das et al., 2020). The aim of this research was to use zeolite/cellulose acetate blended fiber as a reusable, simple-to-prepare adsorbent for erythromycin adsorption. The effects of several parameters, including contact time, concentration effect, temperature effect, and equilibrium and kinetics, on erythromycin adsorption by the composite fiber were studied.

SEM, FT-IR spectroscopy, thermogravimetric analysis, and dynamic scanning calorimetry were used to characterize the zeolite/cellulose acetate fiber.

The novelty of this work is shown by using three different adsorbents which showed very high percentage of removals. Also, theoretical studies were very supportive of the experimental findings.

## Methods and Materials

### Chemicals

The zeolite compounds are containing aluminum and silicon (M_2_/nO.Al_2_O_3_.xSiO_2_.yH_2_O) where *M* can be any one of a number of metals, including sodium, lithium, potassium, calcium, and magnesium. The variable “*n*” stands for the valence of the metal cation and “*y*” for the number of water molecules in the structure of zeolite, according to the Research Foundation at State University of New York (SUNY). Cellulose acetate (C_10_H_16_O_8_) has been purchased from Al Quds Chemicals in Jerusalem. The zeolite chemical composition was included in the MSDS that has been supplied from the manufacturer. Acetone was bought from Guangzhou Chemi. Erythromycin with technical grade of 99% was purchased from Fluka (Fluka Chemie AG, Switzerland). Acetonitrile was purchased from Sigma–Aldrich, United States with analytical grade of more than 99%. The water was of the Milli-Q standard (Millipore, MA, United States).

### Preparation of ZCA Fiber

Wet spinning was used to produce the zeolite/cellulose acetate blend fiber (ZCA); cellulose acetate (6 g) was dissolved in 50 ml of acetone/water solution (6:1, w/w). The zeolite rocks were ground and sieved to achieve an average dimension of approximately 800 mesh.

1.5 g of zeolite is added to the solution and scattered by mechanical stirring. To make a solid filament, the blended solution was spun in a stainless-steel spinner and then protruded into a water coagulation tank. The fiber was taken out of the bath and washed twice with filtered water. Finally, the fiber was dried at 30°C before being cut into very small fragments (Rodchanasuripron et al., 2020).

### Characterization of ZCA Fiber

The scanning electron microscopy (SEM) manufactured by the Hitachi model (S-4700) in Japan was used to study the morphology of ZCA fiber. ZCA fiber was immersed in a liquid nitrogen atmosphere to create a very clean cross section for scanning. The Hitachi S-4700 FE-SEM is a cold field emission high-resolution scanning electron microscope. This SEM permits ultrahigh resolution imaging of thin films and semiconductor materials on exceptionally clean specimens. It is also suitable for polymeric materials. S-4700 is configured to detect secondary and backscattered electrons as well as characteristic X-rays.

The X-Ray diffraction analysis was done using XRD-Shimadzu XD-1 with monochromatized graphite Cu-K alpha (15,418) and a scanning speed of 20°/min. The Bruker Alpha-P spectrophotometer was used to collect the Fourier transform infrared (FTIR) fiber spectrum. FT-IR spectra were reported from 400 to 4,000 cm^−1^ with 32 scans on Nicolet NEXUS-470 FT-IR (America) apparatus and a resolution of 4 cm^−1^.

The Shimadzu UV absorption spectrum of the sample was tested using an 1800 UV-Vis spectrophotometer with UV probe software. The ERY concentration was measured quantitatively using a UV–Vis spectrophotometer (SHIMADZU, UV-1201). The absorbance of the ERY solution was estimated at 481.5 nm, the wavelength at which ERY has the greatest absorbance.

CuK Al radiation was used for X-ray diffraction on the Panalytical X'Pert Pro diffractometer (1.5418 Å) from 2° to 70° (2*θ*), with a scanning rate of 1° per minute. The water intrusion process was also used to determine membrane porosity (Wang et al., 2017; Bagaev et al., 2021).

Thermogravimetric analysis was carried out on DTG 60H equipment (Shimadzu Co., Japan). Around 3.0 mg of adsorbents were heated from 25 to 700°C in the nitrogen atmosphere (50 ml/min) at a temperature of 10 0°C/min. The compounds’ decomposition temperatures were calculated using the first mass loss (percentage) vs. temperature derivative (DTGA) (Güler et al., 2013; Zhang et al., 2014).

### Adsorption Procedure (Import)

**FIGURE 1:**
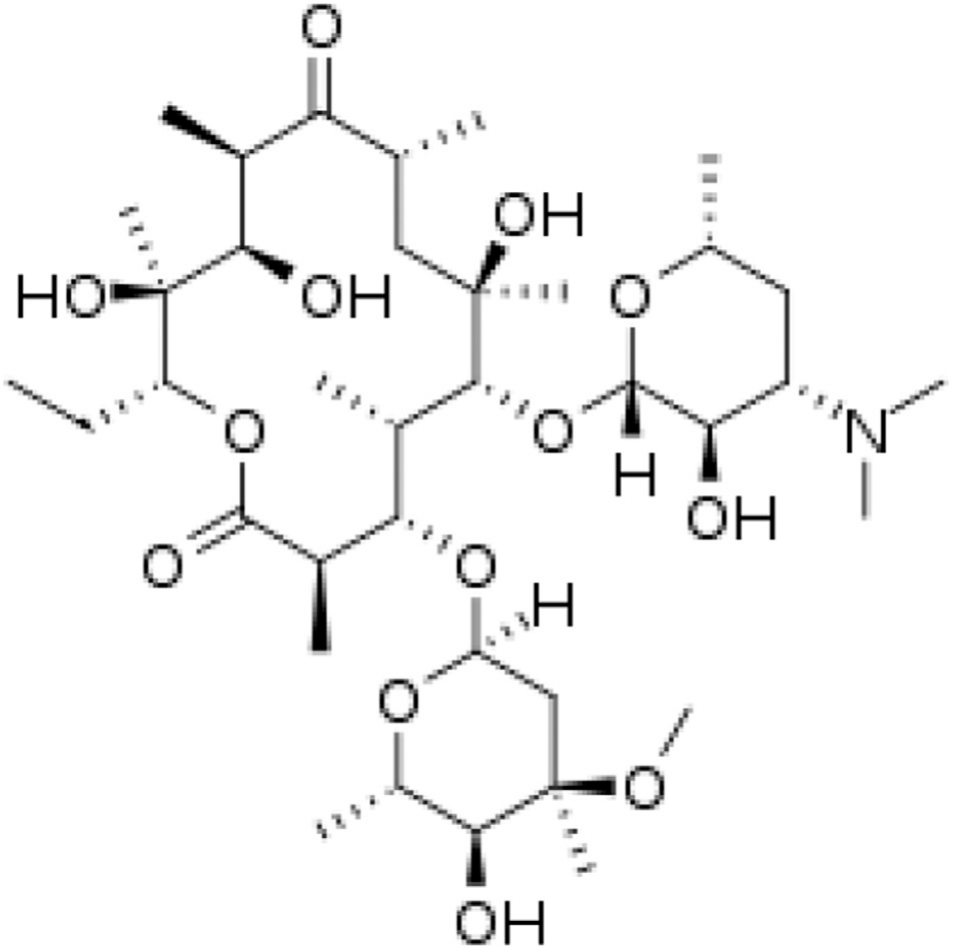
Structure for erythromycin.

Erythromycin [C37H67NO13] with molecular mass of 733.937 g mol^−1^ is an antibiotic used for the treatment of a variety of Fluka. The chemical structure is presented in Figure 1.

To study the adsorption equilibrium experiments, a sample of 10.0 mg of ZCA fiber was used in most of the analysis. Following that, 100 ml of aqueous solutions with varying initial ERY concentrations (10–50 mg/L) were applied and shaken at 200 rpm in an orbital incubator (Gallenkamp, model INR-250). To achieve adsorption equilibrium, the contact time was varied between 5 and 90 min. The other study was performed to see the effect of temperature on the adsorbent activity and efficiency at different temperatures and constant contact time of 30 min, and the temperatures were 25, 35, 45, and 55°C. In each study, a UV-Vis (Varian, model Cary 1E) spectrophotometer (*λ*max: 482 nm) was used to measure ERY equilibrium concentrations using a calibration curve of different concentrations (Jamshaid et al., 2020).

The effect of pH was studied from 2 to 12, and both 0.1 M NaOH and 0.1 M HCl solutions were used to change the pH as required. At 293 K, 100 ml of ERY solution containing 20 mg/L was shook with 10.0 mg of ZCA fiber.

The pH study was carried using a micro pH 2002 Crison pH meter. All equilibrium concentrations of the adsorbed ERY by ZCA were presented using different adsorption parameters; q_e_ (e.g., in mg/g) was calculated using the following equations (Eqs 1, 2) (Abujaber et al., 2018):

$$q_{e}=\frac{V\left(Co-Ce\right)}{W},$$ (1)

$$\%R=\frac{\left(Co-Ce\right)}{Co}*100\;\%,$$ (2)

where q_e_ is the amount (mg g^−1^) adsorbed, C_o_ and C_e_ are the ERY initial and equilibrium concentrations in solution (mg/L), respectively, *W* is the adsorbent dosage (g/L), and *R* percent is the adsorption efficiency coefficient. The kinetic study was done by taking several dosages of ZCA (50, 100, and 150 mg/L). This study’s tested temperatures were 293, 303, and 313 K, with a maximum contact time of 60 min.

### Adsorption Kinetics

Pseudo–first-order and second-order models have been used to model the kinetic effects of ERY adsorption on the surface of ZCA fibers to achieve the control rate structure of adsorption including chemical reactions and mass transfer. As seen in Eq. 3, pseudo–first-order modeling is based on the premise that physical adsorption that occurs during the removal process is the rate-determining step (Azzaoui et al., 2017):

$$\log\left(q_{e}-q_{t}\right)=\log q_{e}-\left(\frac{K_{1}}{2.303}\right)t,$$ (3)

where q_e_ (mg/g) represents the equilibrium adsorbed ERY quantity, q_t_ (mg/g) represents the equilibrium adsorbed ERY quantity at time *t*, and K1 (min^−1^) represents the pseudo–first-order modeling adsorption rate constant (Azzaoui et al., 2017). The modeling of the pseudo–second-order, on the other hand, was based on the assumption that the rate-determining process, as shown by Eq. 4, is chemi-sorption:

$$\frac{t}{q_{t}}=\frac{1}{q_{e}}t+\frac{1}{K_{2}q_{e}^{2}},$$ (4)

where K_2_ (g/mg/min) is used as the pseudo–second-order rate constant. The slope and intercept of a plot of t/q_t_ vs. t are used to calculate the values of q_e_ and K_2_, respectively. ERY was controlled for attachment to the ZCA surface through chemical bond forming in the chemical adsorption process.

### Adsorption Isotherm

Adsorption isotherms usually have data on the distribution of adsorbed molecules in equilibrium between solid and liquid phases. Most experiments used the regression coefficient (*R* ^2^) to assess the best-fitting isotherms. Adsorption equilibrium results were discovered to be more appropriate for two types of Freundlich and Langmuir isothermal models.

The most fundamental model is Langmuir, which assumes that all adsorption sites are equal and autonomous. The tendency of molecules to bind is separate from the neighboring populated sites (Radi et al., 2015). The isotherm of Langmuir can be given by the following equation:

$$\frac{{\text{C}}_{\text{e}}}{{\text{q}}_{\text{e}}}=\frac{1}{{\text{q}}_{\text{m}}}{\text{C}}_{\text{e}}+\frac{1}{{\text{q}}_{\text{m}}{\text{K}}_{\text{L}}}\text{ ,}$$ (5)

where Ce is the ERY equilibrium concentration (mg/L), qe is the sum of ERY adsorbed per gram of the three equilibrium adsorbents (mg/g), and qm is the full potential of monolayer coverage (mg/g) (Radi et al., 2015). The Langmuir isotherm (L/mg) constant is K_L_.

The Freundlich isotherm, on the other hand, demonstrates un-ideal and reversible adsorption. The best representation of heterogeneous structures is preferred. It is possible to approximate Freundlich isotherm by the following equation:

$$\ln q_{e}=\;\frac{1}{n}ln\;C_{e}+lnK_{F},$$ (6)

where qe is the capacity of adsorption, Ce is the ERY concentration at equilibrium, and KF and *n* are constants. K_F_ reflects the capacity of adsorption, whereas *n* reflects the deviation from linearity of adsorption. If *n* = 1, the process of adsorption is linear; if *n* < 1 the process of chemical adsorption; and if *n* > 1, the process of adsorption is favorable. The Langmuir model is limited to monolayer adsorption. The Langmuir model is limited to monolayer adsorption systems, whereas in multilayer systems, the Freundlich model can be used.

### Adsorption Thermodynamics

From the obtained kinetic data, the reaction rate and other thermodynamics parameters can be identified. Nonetheless, the response changes that will happen during the process of adsorption require the determination of the thermodynamic parameters, including entropy [(∆S, kJ/mol), enthalpy, free-energy Gibbs (∆G, kJ/mol), and adsorption changes (∆H, kJ/mol). You can calculate the thermodynamic parameters from the van’t Hoff Eq. 7]:

$$lnK_{d}=-\frac{H^{o}}{RT}+\frac{S^{o}\;}{R}\;,$$ (7)

where the gas constant is *R* (8.314 J/mol/T) and the temperature is T (K). Eq. 8 can be used to calculate the distribution coefficient (Kd) on the adsorbent surface.

$$K_{d}=\frac{V\left(Co-Ce\right)}{W*\text{Ce}}.$$ (8)

Gibbs free energy can be calculated by the following equation:

$$\Delta G^{\text{o}}=–\text{ R}T\text{ ln }K_{D}.$$ (9)

Both ∆H and ∆S can be calculated using both slope and intercept from the van’t Hoff plot of lnK vs. 1/T (Hanbali et al., 2020).

### Computational and Theoretical Study

The geometry optimization of ERY was performed by G09W (frisch et al., 2009) with the Hartree–Fock (Roothaan, 1951; Pople and Nesbet, 1954) method and 6-311G** (Krishnan et al., 1980; McLean and Chandler, 1980) basis set in the gas phase. In theoretical predictions of the chemical reactivity, the Koopmans’ theorem (Koopmans, 1934) is the first essential step to calculate the ionization energy (*I*) and electron affinity (*A*) values *via* the FMO energies.

*I* = −E_HOMO_
*A* = −E_LUMO_

Moreover, the quantum chemical parameters (QCP) (Parr and Pearson, 1983; Pearson, 1986; Pearson, 1988; Parr et al., 1999), which are defined as *χ* “electronic chemical potential,” *η* “global hardness,” *ω* “electrophilicity index,” and ΔN “fractional number of the electrons transferred” in case of B and C systems have contacted each other, and ΔN_max_ “maximum charge transfer capability,” have been also obtained from the *I* and *A* values using the following formula:

$$\chi =-\frac{I+A}{2},$$

$$\text{η}=\frac{I-A}{2},$$

$$\omega =\frac{\mu^{2}}{2\text{η}},$$

$$\Delta N=\frac{\chi_{C}-\chi_{B}}{2\left(\eta_{C}+\eta_{B}\right)},$$

$$\Delta N_{max}=\frac{I+A}{2\left(I-A\right)}.$$

In addition, Gazquez and coworkers introduced two useful parameters to calculate the *ω* ^−^ “the electron-donating power” and *ω* ^+^ “the electron-accepting power” parameters (Gázquez et al., 2007)

$$\omega^{+}\approx {\left(I+3A\right)}^{2}/\left(16\left(I-A\right)\right),$$

$$\omega^{-}\approx {\left(3I+A\right)}^{2}/\left(16\left(I-A\right)\right).$$

Also, the ΔE_back-donation_ “back-donation energy” (Gómez et al., 2006) is a powerful value and defined as the following equation:

$$\Delta \varepsilon_{back-donation}=-\frac{\eta }{4}.$$

In addition, the stabilization energy lowering obtained from the second-order perturbative energy analyses depending on the NBOs “Natural Bon Orbitals” (Foster and Weinhold, 1980; Reed and Weinhold, 1985; Reed et al., 1988) is defined as follows:

$$E^{\left(2\right)}=E_{ij}=qi\frac{{\left(Fij\right)}^{2}}{\left(\varepsilon j-\varepsilon i\right)}.$$

For the molecular system, *qi* states the donor orbital occupancy, *εi* and *εj* are diagonal elements, and *Fij* is the off-diagonal NBO Fock matrix element where “*i*” and “*j*” are the filled and unfilled molecular orbitals.

### Regeneration of Adsorbent

In the field of adsorption process applications, adsorbent regeneration is important. ZCA samples were pre-adsorbed for 12 h at 25°C with 10 ml of 50 mg/L ERY solution, then washed with methanol/acetic acid (v/v, 9:1) until no ERY was present in the eluent, and dried overnight at 50°C. Following that, regenerated materials were redistributed in 10 ml solutions containing an initial concentration of 50 mg/L. The effectiveness of ERY adsorption by regenerated materials has been studied after several adsorption–desorption processes.

## Results and Discussion

### Adsorbent Characterization Results (BET)

Nitrogen adsorption–desorption isotherm measurements were carried at 77 K using a Quantachrome Autosorb AS-1 instrument (United States). The BET specific surface area of ZCA was measured using the data of nitrogen adsorption isotherm at low temperature (Brunauer et al., 1938) and involving the adsorption data at *P/P*_*0*_ of 0.05–0.2 and with 2.47 m^2^/g. The BJH model was used to measure the pore volume and the average pore size as other previous study (Barrett et al., 1951). The pore volume of ZCA sample was determined as 2.45 × 10^−2^ cm^3^/g, and pore diameter was 3.5 nm. The ZCA pore diameter was considered as a mesoporous material as the classification by the Pure and Applied Chemistry International Union (IUPAC) (Foster and Weinhold, 1980).

### Characterization of ZCA Fiber Using SEM

**FIGURE 2:**
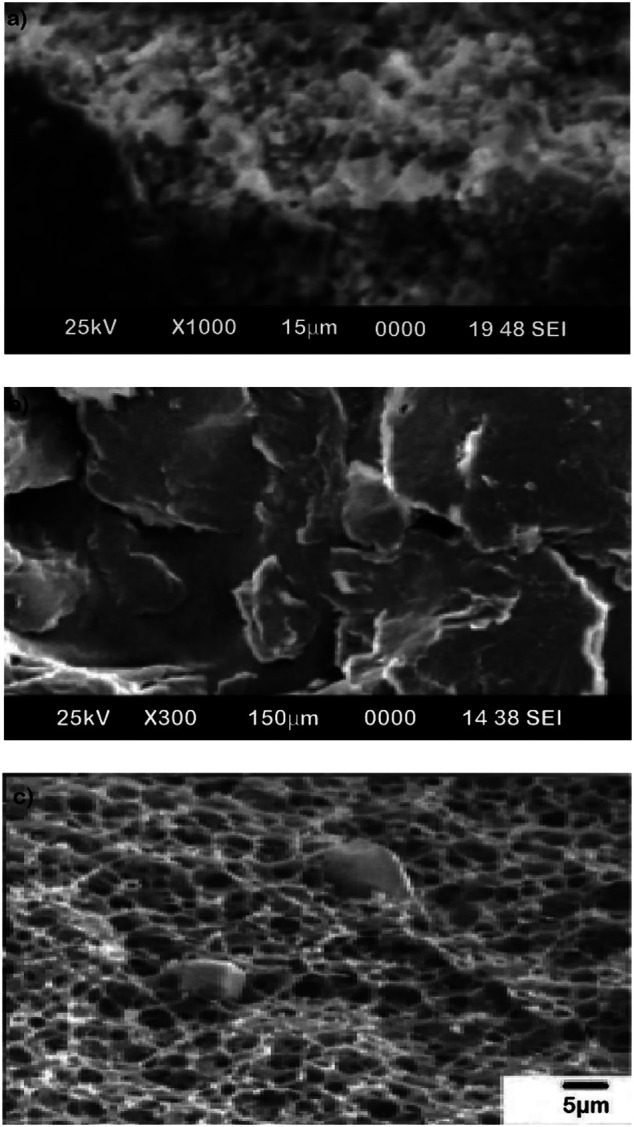
SEM images of **(A)** ZCA fiber, **(B)** zeolite, and **(C)** cellulose acetate (CA).

SEM was used to examine the morphology of ZCA fiber. The surface morphologies and cross-sectional configurations of the ZCA filament are shown in Figure 2. The surface of the ZCA fiber is relatively smooth, as seen in Figure 1, and the diameter of the as-prepared fiber is approximately 250 nm. As seen in the cross-section, the ZCA fiber has a sponge-like appearance. The ZCA fiber is composed of a homogeneous, highly porous material. The ZCA fiber network is embedded with zeolite crystals about 100°m in height. As seen in Figure 2, cellulose acetate serves as a matrix support, and the pore size of the fiber ranges between 5 and 10 m. ERY could rapidly disperse into the pores for contact with the adsorptive sites of the ZCA particles.

The dispersion of zeolite attributed by the silica and aluminum shown in Figures 1B,C indicates that the zeolites were embedded in the cellulose acetate matrix. This is attributed to the interfacial interaction between zeolite and cellulose acetate.

### X-Ray Diffraction Analysis

**FIGURE 3:**
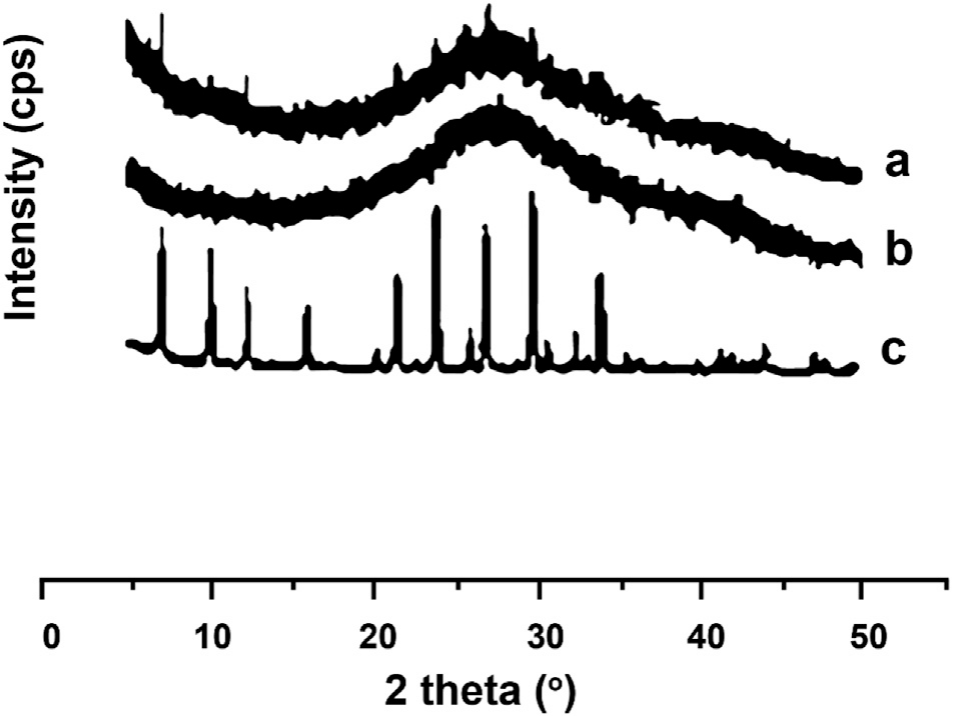
Comparison of X-ray diffractograms of **(A)** zeolite, **(B)** CA, and **(C)** ZCA.

The diffractogram of the synthesized zeolite is identical to JCPDS No. PDF 0038-0241 for LTA form zeolite-A [Na96(AlO2)96(SiO2)96.216H2O] as seen in Figure 3. Furthermore, diffractogram of CA, as shown in the figure, appropriates with a diffractogram reported by Fan et al. (2013), who stated that CA has distinctive angles at 2*θ* of 10° and 13.2°. These two typical angles were also recognized as the crystalline peaks of modified CTA II (Deus et al., 1991). Furthermore, Jayalakshmi et al. (2014) announced that the CA membrane diffractogram had a normal semicrystalline angle at 2 of 9.6° and two crystalline angles at diffraction angles of 20.1° and 26.8°.

The diffractogram of CA membrane in this study was identified as a crystalline peak at 26.8°. Composite membrane also has a crystalline peak at 26.8°. Moreover, the composite membrane has also a weak peak at 10° and 13.2°, indicating the typical peak of CA in different intensities. It was caused by a decreasing crystallinity form in the membrane compared to CA solids. It was reviewed that the CA/ZA membrane has a peak at an angle of 10.3, 12.6, and 16.2, indicating the presence of zeolite-A. Based on the results of the composite membrane diffractograms, it was known that zeolite-A has better dispersity in the CA porous membrane as a filler.

### FT-IR Analysis

**FIGURE 4:**
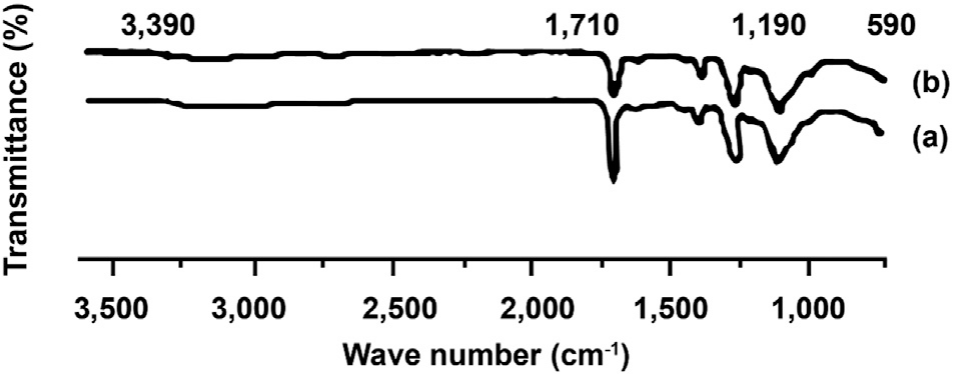
FT-IR for ZCA **(A)** before and **(B)** after adsorption process of ERY.

Figure 4 demonstrates the ZCA fiber FTIR spectrum before and after ERY adsorption. As can be seen, a peak of 600–800 cm^−1^ was observed, which is associated with T-O-T stretching and T-O zeolite bending (Armaroli et al., 2006).

A sharp peak in such regions indicates the presence of zeolite inside the membrane. In addition, the membrane showed a peak in the region of 1,000–1,200 cm^−1^, indicating the interaction between Si-O-Si of zeolite and CA.

Some peaks were also detected at 1,735–1,738 cm^−1^ assigned to carbonyl C=O stretching of CA and broad peak at about 3,400 cm^–1^ assigned to O-H stretching. Furthermore, the absence of new peaks was observed on the membrane after the adsorption process. However, the peak was slightly shifted and the peak intensity decreased. This might be due to the presence of van der Waals force, indicating the physical adsorption between the metal ions and membrane.

### Thermogravimetric Analysis

**FIGURE 5:**
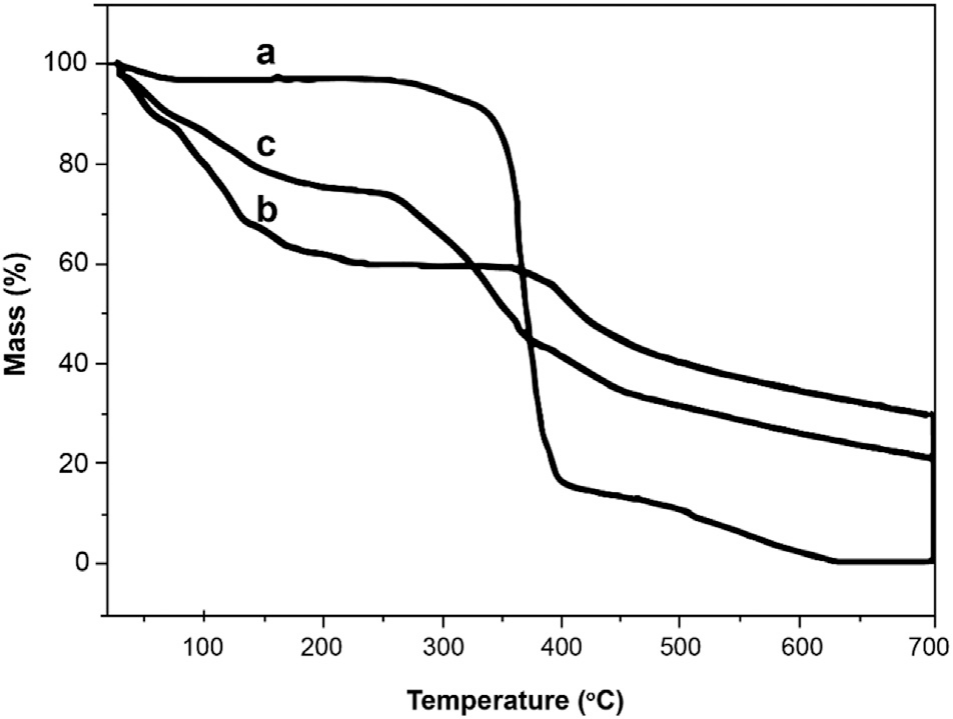
Thermogravimetric analysis (TGA) for **(A)** zeolite\CA (ZCA), **(B)** CA, and **(C)** zeolite.

Thermogravimetric analysis for the three samples, namely, ZCA, cellulose acetate, and zeolite, is presented in Figure 5. From the TGA thermogram obtained for cellulose acetate (CA), there is initially a minor weight loss of about 3% up to 200°C, which is caused from the loss of volatile compounds and the moisture of H_2_O that is bound to the hydrophilic (OH) groups that is bonded in the chain of cellulose acetate chains (Hong et al., 2020).

There are two steps of thermal decomposition: the first phase (300–400°C) which refers to the major loss with a proximate weight loss of 75%, while the second one (400 and 600°C) having a weight loss of 15% is referred to the complete degradation and composition (Hong et al., 2020).

Two levels of mass reduction have been found for zeolite. The first stage was between 30 and 230°C, with a weight loss of 40% which can be due to the loss of H_2_O adsorbed to the material and to the deterioration of certain aluminum and silicate fractions which did not decompose at 400°C during the pyrolysis process. The second stage of zeolite thermal decomposition, starting at 380°C, was caused from the removal of minerals and salts from the material, which has 35% of its initial mass which is considered as its high mineral residue content.

The ZCA fiber thermogram showed three levels of thermal decomposition between 30 and 200°C, 215 and 380°C, and above 380°C. This thermogram showed an intermediate profile in comparison to the CA and zeolite thermograms; that is, for both of the temperature scales of the thermal events referred to above, their mass variations occurred roughly as the sum of the other two thermograms, because the fiber is made up of 50% of the weight of each part. The first process, with a weight loss of approximately 20%, can be attributed mainly to the release of H_2_O from the material due to the presence of zeolite, with the CA mass being practically constant in this temperature range. The second stage of decomposition is probably due to the degradation of the CA chain, with the zeolite mass remaining almost unchanged. The CA mass loss at this stage was 80%. The third and final stage can be due to complete fiber degradation, and part of the fiber has thermal stability lower than CA, with maximum CA losses at 335 and 360°C, respectively.

### Differential Scanning Calorimetry

**FIGURE 6:**
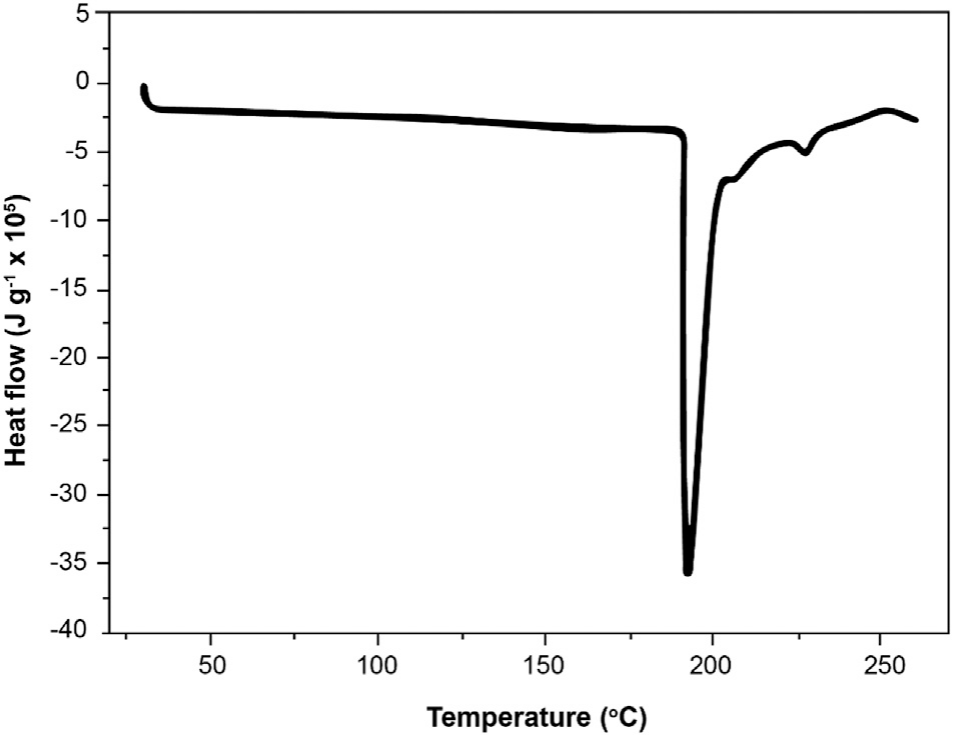
Differential scanning calorimetry (DSC) for the cellulose acetate/zeolite (ZCA) fiber.

The DSC thermogram obtained for the ZCA is shown in Figure 6. The peaks were shown at different temperatures (180, 211, and 225°C). ZCA melting happened at a temperature lower than that of CA melting as indicated by other studies (Gómez et al., 2006). This may have been explained by the fact, that is, the strengthening as well as a lower amount of contacts between the CA chains. Also, the melting enthalpy was 3,600 kJ/g for ZCA. The higher energy involved during the ZCA melting process may be due to water volatilization, since TGA showed large mass loss in this temperature range.

### Adsorption Study

#### Effect of Contact Time

**FIGURE 7:**
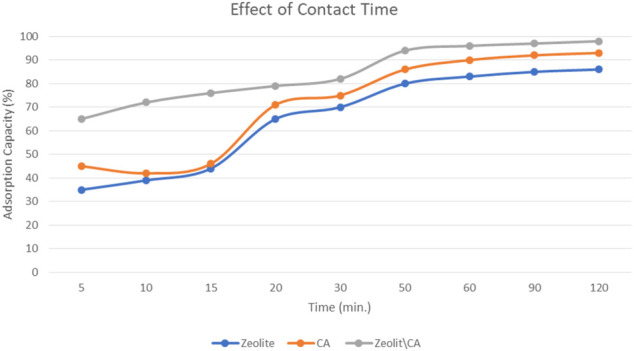
Effect of time on the ERY adsorption onto the three adsorbents.

The effect of equilibrium adsorption time on adsorption efficiency was studied at room temperature close to 25°C. To study that, an initial concentration of ERY of 20 mgL^−1^ and about 20 mg of ZCA adsorbent were used at different time intervals: 15, 30, 45, 60, 75, 90, and 120 min, as shown in Figure 7. The presence of large number of active sites made the adsorption of ERY to the surface of the adsorbents very easy and increased rapidly at an early stage. This process was followed by a slower rise in adsorption. This shows that the complex derivatives formed at the initial stage of adsorption are unstable, resulting in a rapid rate of adsorption. As a result of the presence of hydrogen protons emitted to the oxygen-containing solution on the adsorbent surface beside the presence of hydroxyl and carboxyl groups, this causes a slower adsorption speeds which could be due to a reduction in the driving force of the present adsorption sites. The various efficiencies of adsorption have shown that the absorbents do not show identical morphologies.

#### Effect of Temperature

**FIGURE 8:**
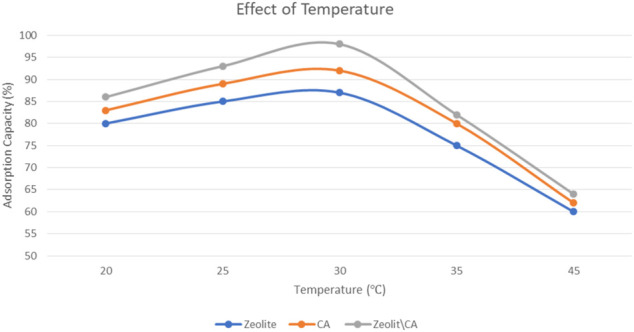
Effect of temperature on ERY adsorption onto the three adsorbents.

Measurements of adsorption were carried out using an adsorbent weight of 20 mg, an initial concentration of 20 mg/L, and a time interval of 60 min. The removal of ERY, controlled by CA, zeolite, and ZCA tests, increased with a rise in temperature from 20 to 45°C, initially indicating an endothermic adsorption mechanism up to 30°C (Figure 8). This could lead to an improvement in the diffusion rate of ERY in the porous structure of the ZCA derivatives, raising the temperature. Due to high temperatures, the adsorption mechanism can include both physical and chemical adsorption, resulting in increased active sites due to bond breakup. The endothermic adsorption process can therefore be attributed to increased pore diameter. Nevertheless, increases in the removal of ERY were controlled with a rise in temperature from 20 to 45°C using CA, zeolite, and ZCA samples, showing a concentration equilibrium between ERY and adsorbents.

#### Effect of ERY Initial Concentration

**FIGURE 9:**
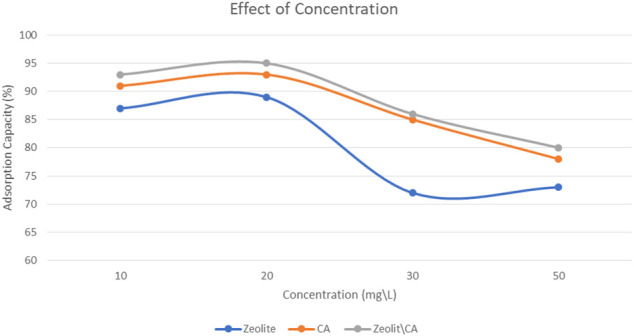
Effect of ERY initial concentration onto the three adsorbents.

Measurements of adsorption at room temperature (25°C) were carried out using separate initial ERY concentrations of 10, 20, 30, and 40 mg/L for 60 min and 20 mg of the three adsorbents, as shown in Figure 9. With an increase in the overall ERY content of up to 20 mg/L, the adsorption process improved and then started to decrease.

The number of interchangeable sites in the adsorbent structures and the ratio of ERY to the three adsorbents were identified as the main factors for the decline in adsorption as initial ERY concentrations increased. The exchangeable sites on the adsorbents are saturated after increasing the ratio of ERY, resulting in a decrease in the efficiency of adsorption. It was observed that the adsorption capacity of adsorbents improved by 5% with an improvement in initial ERY concentrations from 10 to 20 mg/L. This may be the result of the substantial driving force transferred by the ERY concentration in order to defeat the resistance to mass movement between solid and liquid phases.

As seen in Table 1, with reference to the previous studies, the innovation of this study can be summarized as using zeolite/cellulose acetate blended fiber as the first example in the ERY removal literature.

**TABLE 1:** Previous studies on ERY removal from water.

Adsorbent	Optimum condition	Percentage removal (%) or adsorption capacity (q_m_)	Reference
Magnetic activated carbon	Initial ERY concentration of 65 mg L^−1^, sorbent weight of 1.55 g L^−1^, the contact time of 76.25 min, and at the temperature of 35°C	95.125%	Gholamiyan et al. (2020)
Magnetic imprinted polymers (MIPs) from chitosan	Initial ERY concentration of 10 mg L^−1^, and at the temperature of 25°C. pH = 4	Adsorption capacity (q_m_) = 52.32 μmol/g at 15°C	Ou et al. (2015)
Multi-walled carbon nanotubes	Mixing rate of 200 rpm, amount of adsorbent up to 1 g/L, and at the temperature of 75°C	99.4%	Mostafapour et al. (2018)
Porous magnetic graphene (PMG)	pH of 3, contact time of 30 min, initial antibiotic concentration of 200 mg/L, and adsorbent dose of 0.35 g/L	adsorption capacity (q_m_) = 286 mg/g	Fateme et al. (2020)
Fe_3_O_4_/activated carbon/chitosan (MACC: Magnetic activated carbon/chitosan)	15 mg adsorbent, and at the temperature of 20°C	adsorption capacity (q_m_) = 526.31 mg/g	Danalıoğlu et al. (2017)
Amberlite XAD-4	0.002 mg adsorbent at 30°C	adsorption capacity (q_m_) = 358 mg/g	Ribeiro and Ribeiro (2003)
Zeolite/cellulose acetate blend fiber (our study)	Initial ERY concentration of 20 mg L^−1^, the contact time of 60 min, and at the temperature of 30°C	98%	–

### Kinetic Models and Adsorption Isotherms

In this study, the modeling of adsorption kinetics was studied to help and describe the adsorption rate–controlling mechanism.

We studied the adsorption kinetics of ERY using the three adsorbents at initial concentration of 30 mg/L and at 25°C. From this study, the obtained kinetic data were analyzed with the pseudo–first-order (Radi et al., 2015), pseudo–second-order (Hanbali et al., 2020), and intraparticle diffusion using Eqs 3, 4, 10 respectively.

**FIGURE 10:**
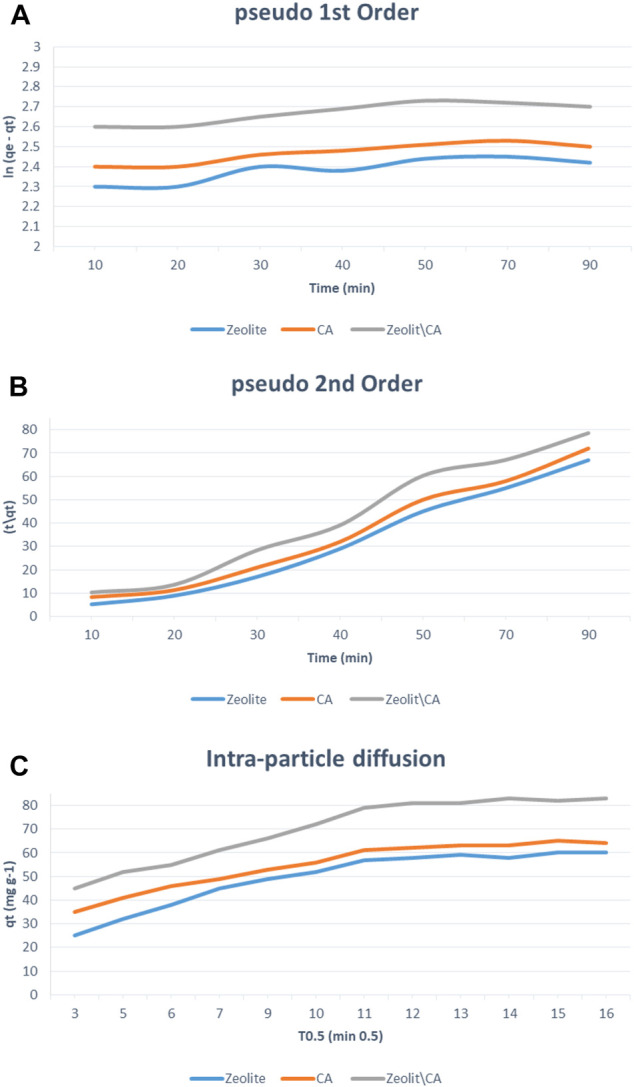
Kinetic models of **(A)** pseudo–first-order, **(B)** pseudo–second-order processes, **(C)** the intraparticle diffusion for the adsorption of ERY by the three adsorbents at different time periods.

As seen in Figure 10, pseudo–second-order modeling showed an improved fit for adsorption calculations relative to pseudo–first-order modeling for all adsorbents.

However, the results obtained in pseudo–first-order modeling were still adequate to define the sorption kinetics of ERY, showing that the surface showed both chemisorption and physi-sorption adsorption processes. The regression coefficient (*R* ^2^) of all adsorbents in the pseudo–second-order is very close to 1 more than the one for the pseudo–first-order. Also, the qe calculated for the three adsorbents in the pseudo–second-order is very close to the experimental one, as shown in Table 2. It has been shown that the pseudo–second-order modeling showed an acceptable match to the adsorption compared to the pseudo–first-order modeling. The movement of ERY from aqueous solution to the adsorbents surfaces might be in different steps, that is, intraparticle diffusion, film diffusion, or both, and that is the rate determining step. The intraparticle diffusion model is shown.

$$q_{t}=Kpi\;t^{1/2}+Ci.$$ (10)

The constant *Ci* represents the boundary layer thickness, and Kpi is a constant. A plot between q_t_ vs. t^1/2^ showed straight line with an appropriate value of correlation coefficient (*R* ^2^) giving the applicability of the intraparticle diffusion model on all three forms of experimental data. For data that match the intraparticle diffusion model, one sees two distinct areas, meaning that two stages are involved in the diffusion process: the external transfer of mass or boundary diffusion of the layer and the intraparticle or micropore diffusion. A greater slope of the first step than the second step suggests a faster adsorption operation, which is due to the more accessible adsorption sites at the initial stage (Hong et al., 2020).

**TABLE 2:** Results of pseudo–first-order, second-order, and intraparticle diffusion kinetic models.

Kinetic model				
	Parameter	Zeolite	CA	Zeolite/CA
1st order	q_exp_	10.16	8.21	9.37
	q_calc_	13.92	17.21	18.23
	K_1_	0.416	0.023	0.098
	*R* ^2^	0.752	0.823	0.788
2nd order	q_calc_	10.62	8.17	9.23
	K^2^	0.85	0.027	0.0335
	*R* ^2^	0.978	0.976	0.98
Intraparticle diffusion	C (mg g^−1^)	14.1	23.9	31.4
	K_id_ (mg g^−1^ h^−0.5^)	4.21	3.76	4.03
	*R* ^2^	0.856	0.898	0.902

### Equilibrium Modeling

**FIGURE 11:**
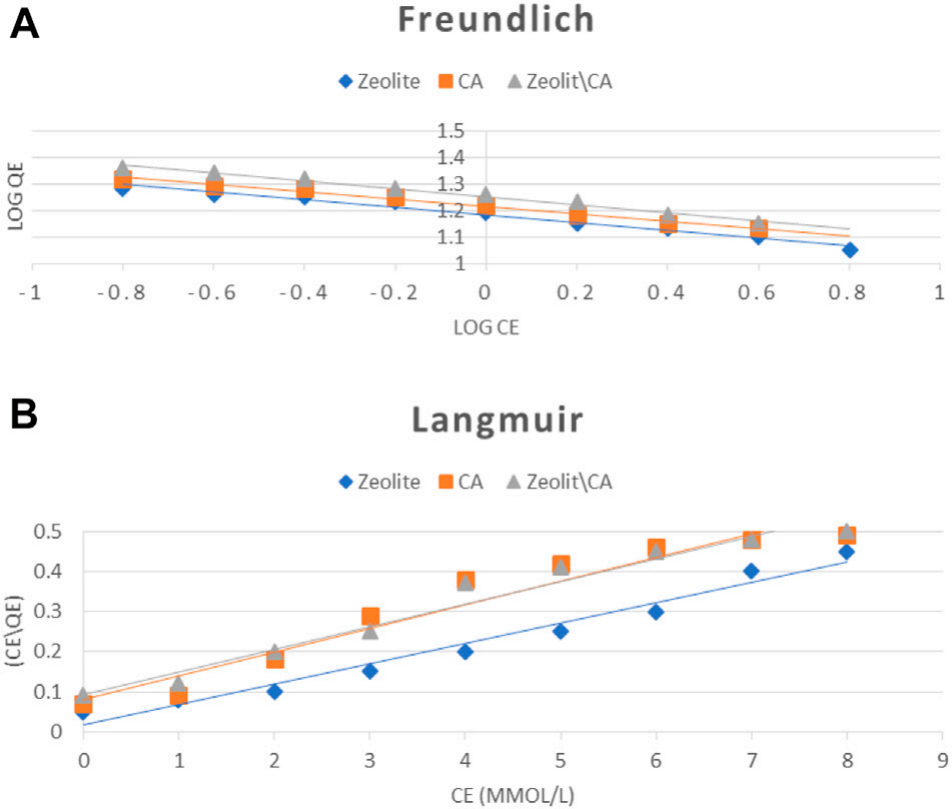
Adsorption isotherm models: **(A)** Freundlich and **(B)** Langmuir models for ERY using three different adsorbents.

Both Langmuir and Freundlich isotherms are the most widely used models for representing equilibrium data of adsorption of ERY onto three adsorbents that were investigated at 25°C for 30 min, with an adsorbent weight of 30 mg/L (Figure 11).

The equilibrium study was carried out in order to understand the mechanism of adsorption process, that is, Langmuir (Hanbali et al., 2020) and Freundlich (Brunauer et al., 1938), which assumes the adsorption of adsorbate as a function of equilibrium concentration. Langmuir isotherm best describes the monolayer adsorption of the solute from solution onto the adsorbent surface having a finite number of active sites present on it. The linear form of the Langmuir isotherm model is shown in Eq. 5.

The results of the models are shown in Table 3. A dimensionless constant R_L_ was calculated using Eq. 11.

$$R_{L}=\frac{1}{\left(1+K_{L}C_{o}\right)},$$ (11)

where Co is the original concentration of ERY (mg/L) and KL is the constant of Langmuir isotherm. The RL value represents adsorption mechanisms that are unfavorable (RL > 1), linear (RL = 1), desirable (1 > RL > 0), or irreversible (RL = 0) (Hong et al., 2020). The R_L_ (0.106) values for ERY in the present study were <1 for the three adsorbents, which indicated favorable adsorption. Freundlich isotherm considers the heterogeneous surface and nonuniform distribution of heat of sorption. It is most favorably studied for description of the multilayer adsorption process (Eq. 6).

**TABLE 3:** Results of Langmuir and Freundlich models for ERY adsorption onto three adsorbents.

Isotherm model				
Model	Parameter	Zeolite	CA	Zeolite/CA
Langmuir	Slope	0.051	0.059	0.056
	Y_int_	0.035	0.023	0.037
	q_m_ (mg g^−1^)	19.61	16.95	17.76
	K_L_	560.22	743.38	477.47
	*R* ^2^	0.971	0.934	0.963
Freundlich	Slope	−0.0287	−0.0281	−0.0305
	Y_int_	1.33	1.35	1.40
	K_f_ (mg^(1−1/*n*)^ g^−1^ L^1/n^)	21.16	22.59	25.24
	*N*	−34.84	−35.59	−32.79
	*R* ^2^	0.975	0.988	0.985

In summary, the studied isotherms were best suited to Langmuir models, which is believed due to the high regression coefficient (*R* ^2^) value (Table 3). It can also be observed that the surfaces of all three adsorbents are homogeneous and that adsorption process occurred mainly in the monolayer system.

### Thermodynamic Study

The adsorption thermodynamics for the adsorption process of ERY onto three adsorbents are displayed in Table 4, in order to understand the nature of ERY adsorption on the three adsorbents using Eqs. 7–9. The three adsorbents showed negative values of ΔH, and the values were −6,200, −8,500, and −9600 kJ/mol for zeolite, CA, and ZCA, respectively, and this shows that the adsorption is exothermic. The positive values of ΔS for ERY on the three adsorbents showed some orderliness on the surfaces of adsorbents. Meanwhile, spontaneous sorption nature of the reaction was depicted by negative values of ΔG, that is, −1.32, −0.1.56, and −1.9 kJ/mol, respectively.

**TABLE 4:** Adsorption thermodynamics for the adsorption of ERY.

Thermodynamic				
Parameter	Temperature	Zeolite	CA	Zeolite/CA
∆S (J mol^−1^)	20	0.08	0.06	0.03
∆H (kJ mol^−1^)	20	−6,200	−8,500	−9,600
∆G (kJ mol^−1^)	20	−0.25	−0.35	−0.62
	25	−1.13	−1.17	−1.36
	30	−1.32	−1.56	−1.9
	35	−2.13	−2.78	−3.26
	45	−2.5	−3.5	−4.1

### Quantum Chemical Studies

The optimized geometry and calculated physical and quantum chemical quantities of ERY are given in Figure 1 and Table 1, respectively. Accordingly, the dipole moment (*D*), polarizability, (*α*), and first-order hyperpolarizability (*β*) values of ERY compound were determined as 4.421 D, 416.124 au, and 169.795 au, respectively. Also, the thermodynamic quantities ΔE, ΔH, and ΔG including the thermal correction were calculated at −2,467.184 au, −2,467.129 au, and −2,467.269 au, respectively. As known well, the vibrational freedom constitutes a remarkable part of the thermal energy as well as the entropy (S) and heat capacity (Cv) for the molecular systems (McQuarrie, 1973; Sandler, 2010; Herzberg, 2013). From Table 5, the thermal energy (ΔE) and vibrational movement contribution to the ΔE (ΔE_vib._) were predicted at 730.238 kcal/mol and 728.461 kcal/mol, respectively. In addition, the Cv and S values of ERY compound were estimated at 207.438 cal/molK and 294.519 cal/molK, respectively, whereas the vibrational part of these quantities were determined at 201.476 cal/molK and 209.940 cal/molK, respectively.

**TABLE 5:** Calculated physical and quantum chemical quantities of ERY at HF/6-311G** level.

QCP		Physical parameters	
HOMO (-I) (eV)	−9.385	DM (debye)	4.421
LUMO (-A) (eV)	0.372	*α* (au)	416.124
ΔE_gap_ (L-H) (eV)	9.757	*β* (au)	169.795
*µ* (eV)	−4.506	ΔE (au)	−2,467.184
*η* (eV)	4.879	ΔH (au)	−2,467.129
*ω* (eV)	2.081	ΔG (au)	−2,467.269
*ω*+ (au)	0.016	ΔE_thermal_ (kcal/mol)	730.238
*ω*− (au)	0.182	ΔE_vib.(thermal)_ (kcal/mol)	728.461
ΔN (eV)	0.032	Cv (cal/molK)	207.438
Δε_back-donat_ (eV)	−1.220	C_vib_ (cal/molK)	201.476
ΔN_max_ (eV)	0.924	S (cal/molK)	294.519
–		S_vib._ (cal/molK)	209.940

In addition, the QCPs are used successfully to assess the reactivity and its selectivity from the simple molecular systems (Serdaroğlu, 2011a; Serdaroğlu, 2011b; Serdaroğlu and Ortiz, 2017; Serdaroğlu et al., 2020) to complex systems (Jacob et al., 2020; Al-Otaibi et al., 2021; Junejo et al., 2021). In this work, the chemical reactivity tendency of ERY was assessed in light of the calculated QCPs and is displayed in Table 5. ΔE_gap_ and *µ* (eV) were determined at 9.757 and −4.506 eV, respectively. As known well, the hardness value is a very helpful parameter to assess the chemical reactivity, especially in the evaluation of the adsorption processes. Hence, it has been the main subject of a series of theoretical investigations (Parr and Pearson, 1983; Berkowitz et al., 1985; Yang and Parr, 1985; Pearson, 1986; Zhou and Parr, 1989; Parr and Chattaraj, 1991; Parr and Gazquez, 1993; von Szentpály et al., 2020; Chaudhary et al., 2021) to be able to calculate it by using the different atomic and/or molecular constants and/or quantities such as ionization energies and electronegativities of the atoms in a specific molecule (Kaya and Kaya, 2015a), and atomic charges (Kaya and Kaya, 2015b). In addition, the molecular hardness has been reported to be able to be used in the theoretical prediction of the lattice energies of the ionic crystals (Kaya and Kaya, 2015c; Kaya et al., 2016; Islam and Kaya, 2018). In this work, the *η* and Δε_back−donat_ values of ERY were calculated at 4.879 and −1.220 eV, respectively. Furthermore, Table 5 displayed that the electron-donating power (0.182°au) of the ERY compound was calculated to be greater than the electron-accepting power (0.016°au), which affirmed that the ERY compound preferred the charge transfer to the metal surfaces. In past, corrosion inhibition efficiency was reported to increase with an increase of the electron-donating ability in case ΔN < 3.6, and *vice versa* for ΔN > 3.6 (Lukovits et al., 2001). According to the ΔN (0.032 < 3.6) and electro-donating power values, the adsorption of ERY toward the studied adsorbents is easily noticed to be actualized *via* the charge transfer from the ERY compound to studied adsorbents.

**FIGURE 12:**
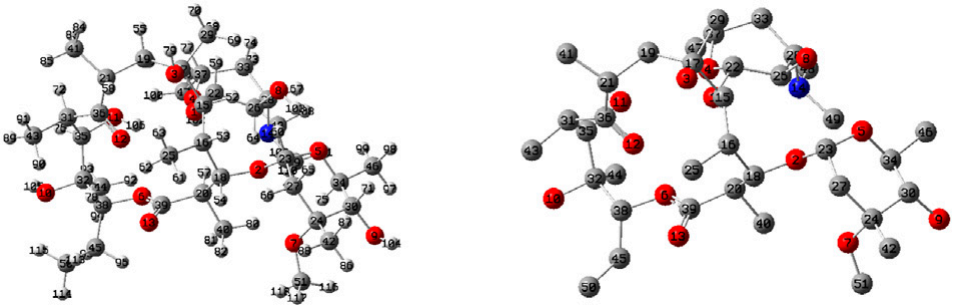
Optimized structures of ERY at HF/6-311G** level (left site with Hs and right side without Hs).

**FIGURE 13:**
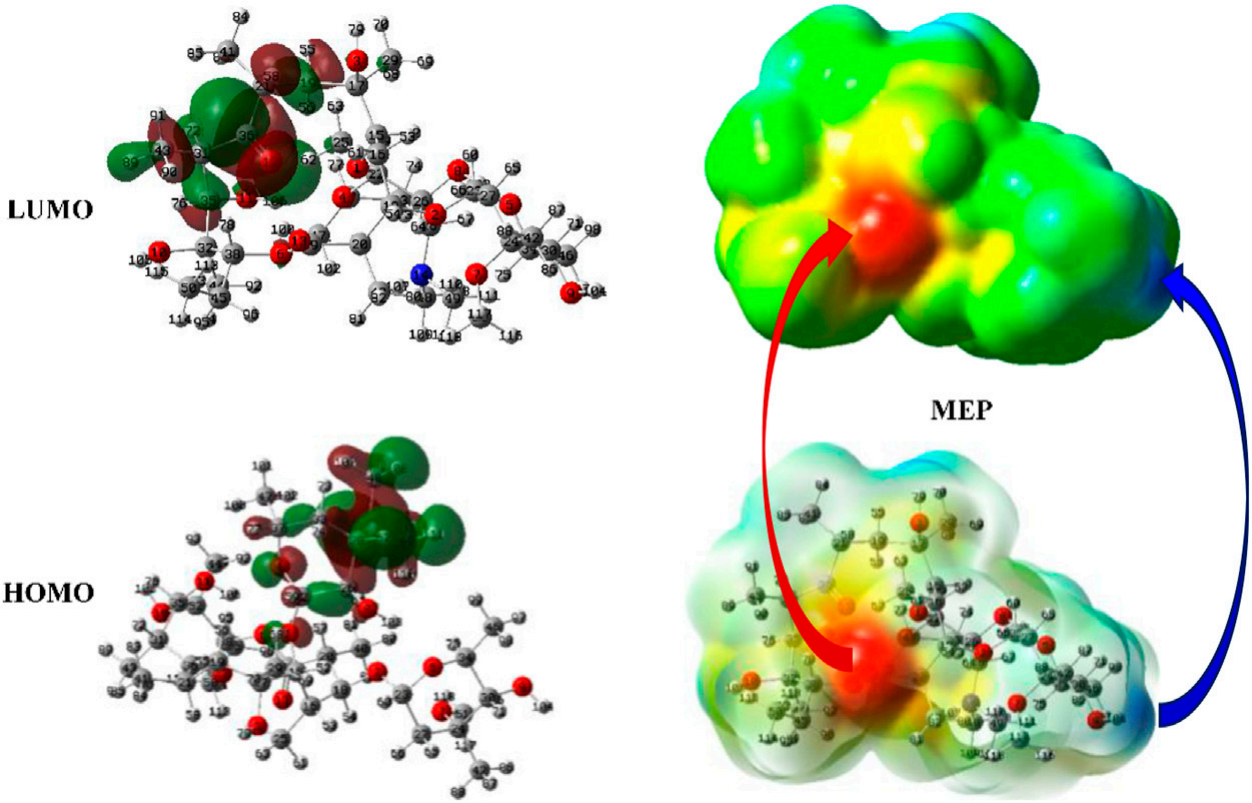
HOMO and LUMO (isoval: 0.02), and MEP (isoval: 0.0004) pilots of ERY at HF/6-311G** level.

Moreover, the possible nucleophilic (HOMO) and electrophilic (LUMO) attack sites of ERY compound are shown in Figure 13. The HOMO density was mostly amplified over the dimethyl amin (-N(CH_3_)_2_) functional and partially be scattered on the oxacyclohexane ring. On the other side, the LUMO broadens on the surrounding of -(C=O)- functional group of ERY. In addition, the MEP graphs displayed the richness of the electron by red color (*V* < 0) and poorness by blue color (*V* > 0) fields of the ERY compound. As expected, the charge transfer zeolites are minerals that contain mainly aluminum and silicon compounds-C=O groups were covered by red color to electrophilic attacks, and the H Atom of the -O-H group was marked by blue color for the nucleophilic attacks. Also, the saturated C- chain of ERY presented neutral attitude for both nucleophiles and electrophiles because it is covered by green color.

The chemical reactivity of many kinds of molecular systems (Mustafa and Serdaroğlu, 2017; Jacob et al., 2020; Serdaroğlu, 2020; Serdaroğlu et al., 2020; Al-Otaibi et al., 2021) has also been clarified by using the results of the second-order perturbative energy analysis. Table 6 summarized the lowering of the stabilization energy, possible interaction types, and the occupancies of both donor and acceptor orbitals. As expected, the mainly saturated structure of the ERY compound, the dominant interactions contributed to *E* ^*(2)*^ (62.33 kcal/mol) were sourced from the charge transfer to anti-bonding orbital П* O13-C39 (ED_j_ = 0.15098e) from nonbonding orbital LP (2) O6 (ED_i_ = 1.84800e). Also, the hyperconjugations due to the charge movement from each filled orbital *σ* C31-C43 and *σ* C31-H72 to unfilled orbital П* C12-O36 were calculated with the *E* ^*(2)*^ of 4.77 kcal/mol and 2.39 kcal/mol, respectively, even if they did not contribute much to the *E* ^*(2)*^. From Table 6, the remaining interactions were due to the anomeric interactions, and the highest energy interactions among them were predicted as the interaction LP (2) O13 (ED_i_ = 1.88472e) → σ* O6-C39 (ED_j_ = 0.07615e) in 42.55 kcal/mol. Similarly, the charge movement from the lone pair of the oxygen atom known as the strong electron-donating of the -C=O group to neighbor orbitals also had great responsibility of energy lowering. Namely, the LP (2) O12 → σ* C21-C36 (*E* ^*(2)*^ = 24.58 kcal/mol), LP (2) O12→ σ* C31-C36 (*E* ^*(2)*^ = 24.89 kcal/mol), and LP (2) O13 → σ* C20-C39 (*E* ^*(2)*^ = 24.31 kcal/mol) interactions also had significant roles in the lowering of the stabilization energy. As known well, the -NH2 group also has strong capability of electron-donating. From Table 6, the charge movement from the N atom of the -NH2 group to each of σ* C28-H67, σ* C48-H108, and σ* C49-H111 interactions was calculated with the energy of 10.72, 10.83, and 10.74 kcal/mol, respectively. Here, it can be considered that these interactions have significant responsibility of the possible intermolecular interactions due to the charge movement that existed in a molecular system, affecting the polarity distribution on the surface.

**TABLE 6:** NBO results for ERY at HF/6-311G** level.

Donor (i)	ED_i_/e	Acceptor (j)	ED_j_/e	E^(2)^/kcal/mol	E(j)-E(i)/a.u	F(i.j)/a.u
*σ* C31-C43	1.97272	П* C12-O36	0.04962	4.77	1.06	0.064
*σ* C31-H72	1.96974			2.39	0.92	0.042
LP (2) O1	1.94227	σ* C15-H17	0.04194	9.47	1.08	0.091
		σ* C22-C26	0.04338	9.64	1.11	0.093
LP (2) O2	1.94036	σ* C18-H54	0.02444	8.56	1.18	0.090
		σ* C23-C27	0.03360	9.06	1.10	0.090
		σ* C23-H60	0.03251	9.90	1.15	0.096
LP (2) O3	1.96470	σ* C17-C19	0.03290	10.64	1.12	0.097
LP (2) O4	1.93923	σ* C22-C26	0.04338	9.30	1.11	0.091
		σ* C37-H77	0.02955	11.13	1.15	0.102
LP (2) O5	1.93444	σ* O2-C23	0.04266	15.27	1.12	0.117
LP (2) O6	1.84800	σ* C38-C45	0.02263	6.57	1.12	0.079
		П* O13-C39	0.15098	62.33	0.74	0.192
LP (2) O7	1.94152	σ* C24-C30	0.04813	10.84	1.10	0.098
		σ* C51-H117	0.01658	9.50	1.12	0.093
LP (2) O8	1.96521	σ* C26-C28	0.03160	9.21	1.11	0.090
LP (2) O9	1.96742	σ* C24-C30	0.04813	9.41	1.13	0.092
LP (2) O10	1.96687	σ* C32-C35	0.04718	8.51	1.10	0.087
LP (2) O11	1.96259	σ* C31-C35	0.03166	9.18	1.10	0.090
LP (2) O12	1.91457	σ* C21-C36	0.05236	24.58	1.10	0.148
		σ* C31-C36	0.05427	24.89	1.11	0.149
LP (2) O13	1.88472	σ* O6-C39	0.07615	42.55	1.13	0.197
		σ* C20-C39	0.05115	24.31	1.08	0.147
LP (1) N14	1.90376	σ* C28-H67	0.02912	10.72	1.11	0.099
		σ* C48-H108	0.02251	10.83	1.07	0.098
		σ* C49-H111	0.02255	10.74	1.07	0.097

### Desorption Study

**FIGURE 14:**
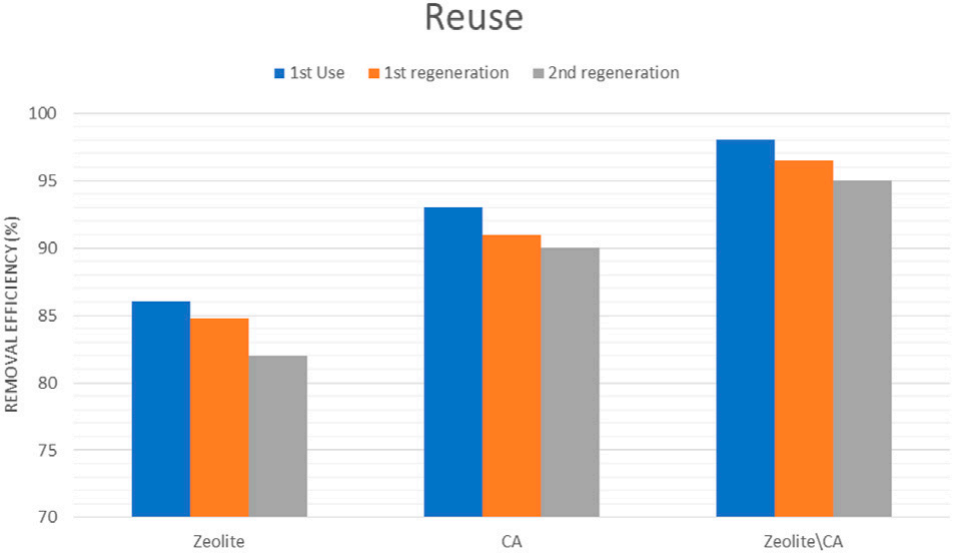
Three cycles for each adsorbent showing excellent reuse.

The stability and reusability of the three adsorbents are especially critical for widespread applications. The adsorption–desorption recycling test, as shown in Figure 14, was used to investigate the adsorbents’ stability further. The adsorbents were washed twice with ethanol after each run and then reused for the next stage of adsorption (Jodeh et al., 2018; Gholamiyan et al., 2020). The findings show that there is no significant loss of adsorption site after three runs, showing that the three adsorbents are more reliable. After the first three regeneration cycles, the adsorption efficiency loss of the three adsorbents to ERY was only about 5.04%. The result was attributed to the reduction of the binding sites in imprinted polymer matrix during regeneration cycles (Barrett et al., 1951). Therefore, the three adsorbents can be reused at least three times without significantly decreasing their adsorption capacities.

## Conclusion

With an increase in the number of studies and research on the fate of pharmaceuticals, personal care products, and their environmental effects on human beings, many researches have been published. As the population and economies grow, numerous antibiotics are increasingly being used in bio-manufacturing, livestock farming, and pharmaceutical industries.

The QCPs revealed that the adsorption of ERY toward the studied adsorbents actualize *via* the charge transfer from the ERY compound to studied adsorbents, because of the ΔN (0.032 < 3.6) and electro-donating power values. The MEP plots pointed out that the -C=O groups were covered by red color to electrophilic attacks and the H Atom of the -O-H group was marked by blue color for the nucleophilic attacks. The NBO analysis of ERY indicated that the anomeric and hyper-conjugative interactions have chief responsibility of the possible intermolecular interactions because of the charge movement affecting the polarity distribution on the surface.

The three adsorbents zeolite, cellulose acetate, and ZCA were used to study the removal of ERY from aqueous liquid prepared in the lab. Several characterizations were done on both the adsorbents and ERY including SEM, XRD, FTIR, and TGA. A brief summary of the results was shown in Abstract, and more details about the results were presented in the Results section.

In summary, the three adsorbents showed very high removal efficiency and reached more than 98% using the fiber. Those adsorbents showed very high reusability, and this will save a lot of money and protect environment.

## Data Availability

The original contributions presented in the study are included in the article/Supplementary Material; further inquiries can be directed to the corresponding authors.
